# Buying time! VA-ECMO as a bridge to successful reoperative cardiac surgery in a case of traumatic tricuspid valve injury

**DOI:** 10.1093/jscr/rjad597

**Published:** 2023-10-31

**Authors:** Abiah Jacob, Donatus K Okafor, Sanjeev Bhattacharyya, Kit Wong

**Affiliations:** Barts Heart Centre, St.Bartholomew’s Hospital, West Smithfield, EC1A 7BE London, United Kingdom; Barts Heart Centre, St.Bartholomew’s Hospital, West Smithfield, EC1A 7BE London, United Kingdom; Barts Heart Centre, St.Bartholomew’s Hospital, West Smithfield, EC1A 7BE London, United Kingdom; University College London Hospital, 235 Euston Rd., NW1 2BU London, United Kingdom; Barts Heart Centre, St.Bartholomew’s Hospital, West Smithfield, EC1A 7BE London, United Kingdom

**Keywords:** extra corporeal life support, VA-ECMO, traumatic cardiac injury, traumatic tricuspid valve injury, acute cardiogenic shock, case report

## Abstract

Cardiac surgery performed on patients in cardiogenic shock is associated with a high mortality and morbidity. Preoperative Extra Corporeal Membrane Oxygenation (ECMO) in cardiogenic shock gives critically-ill patients a chance for surgical intervention and is associated with better surgical outcomes. We present a 29-year-old male who had a ventricular septal defect closure as a child and presented with multi-organ injuries following polytrauma. He was in cardiogenic shock despite maximal inotropic support. Transesophageal echocardiography demonstrated torrential tricuspid regurgitation (TR) from a flail tricuspid valve (TV) leaflet as the cause of cardiogenic shock. He was stabilized on Veno-Arterial ECMO and underwent reoperative cardiac surgery. Intra-operatively, the anterior leaflet of his TV and its papillary muscle was detached from the right ventricle. He had a successful tissue TV replacement. Early surgery was indicated to treat right ventricular failure due to torrential TR, but due to his restricting non-cardiac injuries, ECMO was successfully used as a short-term support strategy and as a bridge to definitive surgery.

## Introduction

The advancement of extracorporeal life support (ECLS) techniques has added a new dimension to the management of acute cardiac failure in adult patients who fail conventional treatment. Mechanical circulatory support (MCS) with Venoarterial Extra Corporeal Membrane Oxygenation (VA-ECMO) utilized in a traditional central or peripheral fashion or in a temporary ventricular assist device configuration may stabilize patients with decompensated cardiac failure by allowing time for recovery, decision-making, and bridging to implantation of a long-term MCS device and on occasion heart transplantation. This therapy provides a range of options to multidisciplinary teams who are involved in the time-sensitive care of complex patients [1]. Cardiac surgery performed on patients in cardiogenic shock is associated with a high mortality. Preoperative ECMO in cardiogenic shock gives critically-ill patients a higher chance for surgical intervention and is associated with better surgical outcomes [2]. We report the only case of VA-ECMO utilized as a bridge to reoperative cardiac surgery in a patient with traumatic tricuspid valve (TV) injury and acute cardiogenic shock.

## Case presentation

**Figure 1:**
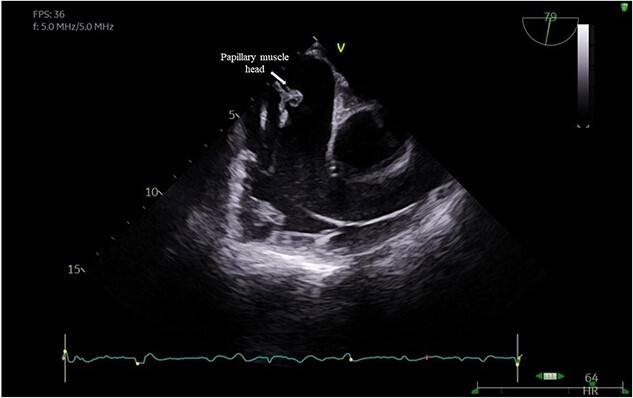
Flail anterior leaflet with associated papillary muscle head.

**Figure 2:**
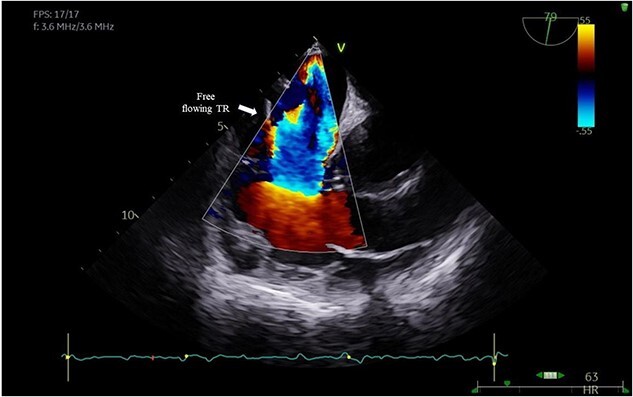
Colour Doppler showing free flowing TR.

A 29-year-old man with a history of ventricular septal defect closure as a child, presented with severe multi-organ injuries after being run down by two cars. He was intubated on the scene by the hospital emergency medical services before being taken to the emergency department for haemodynamic stabilization and multiple imaging procedures. He suffered extensive bilateral degloving scalp lacerations and a haematoma due to bleeding from his left temporal artery. Computed tomography scan revealed bilateral multilevel rib fractures, a left-sided flail segment, bilateral haemopneumothoraces, and multifocal lung contusions, for which bilateral intercostal chest drains were inserted. Moreover, he had a shallow pneumopericardium, a subcapsular left renal haematoma, an extraperitoneal pelvic hematoma, displaced right-sided superior and inferior pubic ramus fractures as well as slight diastasis of the left sacroiliac joint. Although maximum resuscitation measures were taken with adequate fluid and blood product resuscitation, he remained persistently hypotensive and had worsening haemodynamics despite maximal inotropic support. A transoesophageal echocardiogram demonstrated a flail anterior leaflet of the TV and ruptured papillary muscle head (Fig. 1) with torrential tricuspid regurgitation (TR) and high right atrial (RA) pressures (Fig. 2).

Peripheral VA-ECMO via the femoral vessels was instituted for haemodynamic stabilization and as a bridge to cardiac surgery. He was then transferred to our centre where consensus was reached to perform an emergency TV replacement 2 days later. Although high risk, the timing of his surgery was based on his stable haemodynamics which offered an optimal window to give him the best chance at survival, since further delay would lead to either intensive care or ECMO related complications such as bleeding, haemolysis, infection, ischaemia, and circuit problems.

**Figure 3:**
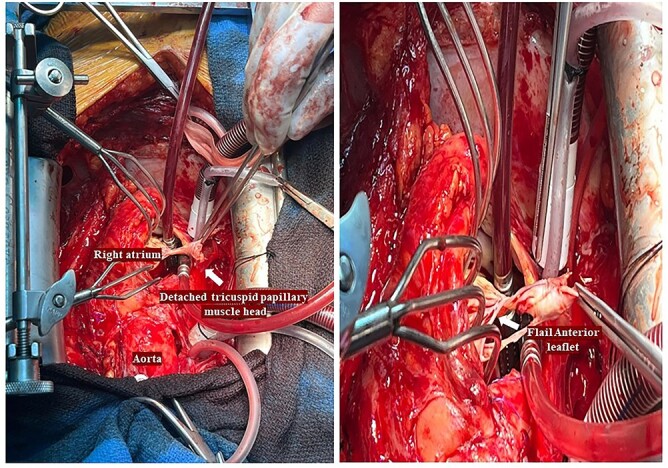
Intraoperative pictures demonstrating a flail anterior leaflet attached to the ruptured head of the papillary muscle.

He had a redo-median sternotomy. Cardiopulmonary bypass was instituted through aortic cannulation connected to the bypass machine via a bifurcated arterial line along with the femoral ECMO cannula. The SVC and IVC were cannulated before opening the right atrium. The TV was inspected which revealed a flail anterior leaflet, attached to the ruptured head of the papillary muscle (Fig. 3).

**Figure 4:**
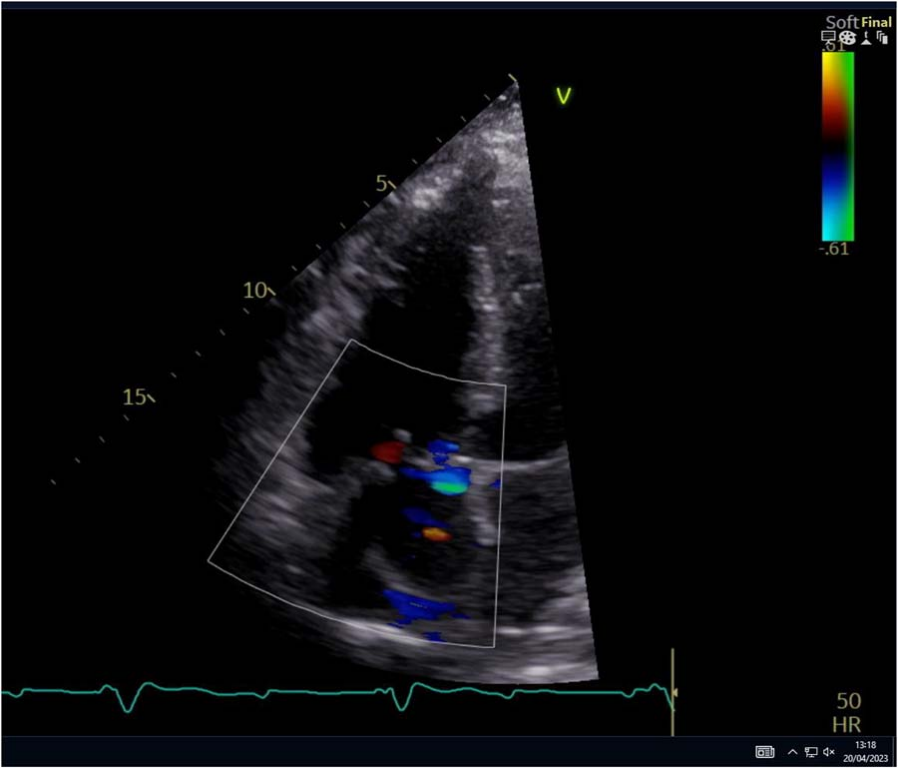
Post-operative ECHO showing a well seated TV.

The leaflet was excised and a 27 mm Mitral Magna Ease tissue valve was implanted. He was successfully weaned off cardiopulmonary bypass and decannulated off ECMO in sinus rhythm. Post-operative ECHO showed a well seated and functioning TV prosthesis (Fig. 4).

Following surgery, his collateral skeletal injuries were addressed after being transferred to a specialist trauma centre. After physiotherapy and rehabilitation, safe mobility was restored and he was discharged home. On follow-up, he is making substantial progress and currently receiving outpatient multidisciplinary care.

## Discussion

We present the only case reported of the utilization of VA-ECMO as a bridge to redo- cardiac surgery in a patient with acute cardiogenic shock due to traumatic tricuspid valve injury (TTI). Blunt cardiac injuries are often fatal and are associated with a much higher mortality versus other organ systems. TV injury is a rare complication of blunt cardiac trauma. The presentation may be subtle since TR can be tolerated and patients may be asymptomatic after trauma and present with the diagnosis months or years later. Currently, it is more frequently reported due to better diagnostic modalities. Early diagnosis and treatment are paramount because untreated tricuspid injury can lead to right ventricular dysfunction, heart failure, and eventually, end-organ damage [3, 4].

The mechanisms of injury include direct compression, decompression, or acceleration/deceleration forces on the thorax and indirect compression from the upper abdomen and limbs causing increased intracardiac pressures [5]. When compressed between the sternum and spine, the right ventricle (RV) is predisposed to injury. The synergy of a closed TV, isovolumetric contraction of the RV, and increased intracardiac pressures generate large traction forces on the valvular and subvalvular apparatus, causing valvular injury [6]. In patients with TTI, common lesions involving the TV are: chordal rupture, papillary muscle rupture, and leaflet rupture [7]. Our patient had a flail anterior leaflet with papillary muscle rupture. There is controversy regarding the optimal timing of surgical intervention for TR. As per 2021 ESC/EACTS Guidelines for the management of valvular heart disease, in severe primary TR, surgery is not only recommended in symptomatic patients but should also be considered in selected asymptomatic or mildly symptomatic patients when RV dilatation or decline in RV function is observed. Although these patients respond well to diuretic therapy, delaying surgery is likely to result in irreversible RV damage, organ failure, and poor results of late surgical intervention [8]. It is hypothesized, that delaying surgery could cause atrophy of the involved leaflets, papillary muscles and chordae tendineae, precluding valve repair [9]. Our patient had a low cardiac output state due to torrential TR with early signs of right ventricular strain. Although urgent surgery was indicated due to his haemodynamic instability, his non-cardiac concomitant injuries may have resulted in a higher risk of complications. In an effort to stabilize his Blood Pressure, VA-ECMO was instituted enabling safe transfer to a cardiac unit and eventual successful cardiac surgery.

VA-ECMO is a powerful therapy used to stabilize patients with haemodynamic compromise, with or without respiratory failure. Although it does not cure the underlying condition, it can be employed:as a bridge to recovery or wean by providing haemodynamic support whilst the heart recovers either spontaneously or with treatment; as a bridge to decision in determining end-organ damage reversibility; and as a bridge to bridge by achieving a period of temporary stability until more definitive mechanical support or cardiac replacement therapy such as transplant is employed [10]. An emerging role of ECMO is in stabilizing patients with haemodynamic compromise and multi-organ dysfunction prior to definitive cardiac surgery [11]. Few reasons for the utility of ECMO in this role and the rationale for delaying definitive surgery are: correction of end-organ dysfunction, correction of coagulopathies, trauma and time for transfer to a tertiary centre as seen in our case [11]. 2021 ESC Guidelines for acute heart failure state that in patients presenting with cardiogenic shock, short-term MCS may be necessary to augment cardiac output and support end-organ perfusion and must be considered as either bridge to bridge, bridge to decision, or as a bridge to recovery [12]. Better outcomes are achievable when high-risk patients are bridged with ECMO to definitive cardiac surgery [13]. Preoperative ECMO is a useful modality to allow haemodynamic stability and organ recovery before definitive cardiac surgery. This case highlights the benefits of ECMO in acute cardiogenic shock due to traumatic cardiac injury. This is the only case reported of VA-ECMO harnessed in such a setting. Early surgery was indicated to treat right ventricular failure due to severe torrential TR, but due to his restricting non-cardiac injuries, ECMO was successfully utilized as a short-term support strategy and as a bridge to definitive redo cardiac surgery.

## Supplementary Material

Ruptured_TV_rjad597

## Data Availability

The data underlying this article are available within the article.

## References

[1] Shekar K, Mullany DV, Thomson B, Ziegenfuss M, Platts DG, Fraser JF. Extracorporeal life support devices and strategies for management of acute cardiorespiratory failure in adult patients: a comprehensive review. Crit Care 2014;18:219. 25032748 10.1186/cc13865 PMC4057103

[2] Abuharb MYI, Ran D, Jubing Z, Taoshuai L, Haiming D, Xiaotong H, et al. Surgical outcomes in cardiogenic shock patients with preoperative extracorporeal membrane oxygenation (ECMO). J Cardiothorac Surg 2021;16:214. 34344398 10.1186/s13019-021-01542-7 PMC8329613

[3] Longfellow E, Aberle C, Lamelas J, Fabbro M 2nd, Johnson E, Yu S, et al. Traumatic injury of the tricuspid valve-navigating the challenges in diagnosis and management. J Cardiothorac Vasc Anesth 2022;36:906–14. 34226110 10.1053/j.jvca.2021.05.049

[4] Conaglen PJ, Ellims A, Royse C, Royse A. Acute repair of traumatic tricuspid regurgitation aided by three-dimensional echocardiography. Heart Lung Circ 2011;20:237–40. 21169058 10.1016/j.hlc.2010.11.004

[5] Kleikamp G, Schnepper U, Körtke H, Breymann T, Körfer R. Tricuspid valve regurgitation following blunt thoracic trauma. Chest 1992;102:1294–6. 1395792 10.1378/chest.102.4.1294

[6] Banning AP, Durrani A, Pillai R. Rupture of the atrial septum and tricuspid valve after blunt chest trauma. Ann Thorac Surg 1997;64:240–2. 9236372 10.1016/s0003-4975(97)00275-0

[7] Zhang Z, Yin K, Dong L, Sun Y, Guo C, Lin Y, et al. Surgical management of traumatic tricuspid insufficiency. J Card Surg 2017;32:342–6. 28543789 10.1111/jocs.13156

[8] Vahanian A, Beyersdorf F, Praz F, Milojevic M, Baldus S, Bauersachs J, et al. 2021 ESC/EACTS guidelines for the management of valvular heart disease. Eur Heart J 2022;43:561–632. Erratum in: Eur Heart J. 2022 Feb 18. 34453165 10.1093/eurheartj/ehab395

[9] Ma WG, Luo GH, Sun HS, Xu JP, Hu SS, Zhu XD. Surgical treatment of traumatic tricuspid insufficiency: experience in 13 cases. Ann Thorac Surg 2010;90:1934–8. 21095339 10.1016/j.athoracsur.2010.07.081

[10] Guglin M, Zucker MJ, Bazan VM, Bozkurt B, El Banayosy A, Estep JD, et al. Venoarterial ECMO for adults: JACC scientific expert panel. J Am Coll Cardiol 2019;73:698–716. 30765037 10.1016/j.jacc.2018.11.038

[11] Wallinder A, Pellegrino V, Fraser JF, McGiffin DC. ECMO as a bridge to non-transplant cardiac surgery. J Card Surg 2017;32:514–21. 28672423 10.1111/jocs.13172

[12] McDonagh TA, Metra M, Adamo M, Gardner RS, Baumbach A, Böhm M, et al. 2021 ESC guidelines for the diagnosis and treatment of acute and chronic heart failure. Eur Heart J 2021;42:3599–726. Erratum in: Eur Heart J. 2021 Oct 14. 34447992 10.1093/eurheartj/ehab368

[13] Dobrilovic N, Lateef O, Michalak L, Delibasic M, Raman J. Extracorporeal membrane oxygenation bridges inoperable patients to definitive cardiac operation. ASAIO J 2019;65:43–8. 29240627 10.1097/MAT.0000000000000741

